# Alleviative Effect of *Ficus formosana* Extract on Peripheral Neuropathy in Ovariectomized Diabetic Mice

**DOI:** 10.3390/plants12213774

**Published:** 2023-11-05

**Authors:** Chih-Yuan Ko, Chung-Hsin Wu, Thi Kim Ngan Nguyen, Li-Wen Chen, James Swi-Bea Wu, Wen-Chung Huang, Szu-Chuan Shen

**Affiliations:** 1 Department of Clinical Nutrition, The Second Affiliated Hospital of Fujian Medical University, Quanzhou 362000, China; yuanmomoko@gmail.com; 2 School of Public Health, Fujian Medical University, Fuzhou 350122, China; 3 School of Medical Technology and Engineering, Fujian Medical University, Fuzhou 350122, China; 4 School of Life Science, National Taiwan Normal University, Taipei 10617, Taiwan; megawu@ntnu.edu.tw; 5 Graduate Program of Nutrition Science, National Taiwan Normal University, Taipei 10617, Taiwan; marynguyen@ntnu.edu.tw (T.K.N.N.); lynn850923@gmail.com (L.-W.C.); 6 Graduate Institute of Food Science and Technology, National Taiwan University, Taipei 10617, Taiwan; jsbwu@ntu.edu.tw; 7 Graduate Institute of Health Industry Technology, Chang Gung University of Science and Technology, Taoyuan 33303, Taiwan; wchuang@mail.cgust.edu.tw

**Keywords:** *Ficus formosana*, diabetic peripheral neuropathy, sciatic nerve, anti-inflammation, oxidative damage

## Abstract

In diabetes mellitus, *Ficus formosana* has been reported to ameliorate blood sugar levels and inhibit inflammation through its polyphenol and flavonoid contents. However, its effect on diabetic peripheral neuropathy (DPN) remains unknown. This study aimed to investigate the effect of *Ficus formosana* extract (FFE) on DPN in ovariectomized diabetic mice. Ovariectomized female C57BL/6J mice fed a high-fat diet plus streptozotocin injections to induce type 2 diabetes were orally administered FEE at 20 or 200 mg/kg BW daily, for 6 weeks. To evaluate the pain responses in the paws of the mice, a von Frey filament test and a thermal hyperalgesia test were performed. Additionally, the intraepidermal and sciatic nerve sections were examined, along with an assessment of inflammation- and pain response-related mRNA expression in the paws of the mice. The results showed that the oral administration of both 20 and 200 mg/kg BW FEE significantly alleviated the hypersensitivity of the paw and the abnormal proliferation and rupture of the C fiber, and reduced the mRNA expression of interleukin-1β, interleukin-6, interferon-γ, cyclooxygenase-2, and voltage-gated sodium channel 1.8 in the sciatic nerve of ovariectomized diabetic mice. We propose that FFE ameliorates peripheral neuropathy by suppressing oxidative damage in ovariectomized diabetic mice.

## 1. Introduction

More than 460 million individuals suffer from diabetes mellitus (DM) globally, of whom, 35% live in the Western Pacific Region [1]. Currently, 4 million individuals die annually owing to DM and its comorbidities [2]. DM can lead to several complications, including diabetic peripheral neuropathy (DPN). Hyperglycemia can cause oxidative stress and peripheral nerve damage, subsequently leading to DPN, which is characterized by skin hypersensitivity, including sensations of crawling insects and pain. In some patients with DPN, severe sensation and mobility impairments can occur and consequently result in death due to the infection caused by foot ulcers [3,4].

In women, menopause and pregnancy can cause hormonal imbalances, which can subsequently lead to insulin resistance and blood sugar level fluctuations. Furthermore, in women, estrogen has been reported to diminish neuroprotective effects and further increase peripheral nerve damage risk [5,6,7]. Moreover, hyperglycemia has been reported to induce oxidative stress, advanced glycation end product formation, glucose oxidation, and polyol pathway activation, subsequently leading to reactive oxygen species (ROS) generation and triggering immune cascades, thereby resulting in inflammation and neural structure/function alterations in the human body [3,4]. Consequently, chronic pain syndrome is attributed to the demyelination, axonal degeneration, and impaired neuronal function of unmyelinated C fibers [8,9].

*Ficus formosana* Maxim (FF) is a ficus plant in the Moraceae family that contains abundant phytochemicals, particularly flavonoids and phenolics, including carpachromene, norartocarpetin, steppogenin, apigenin, 6-prenylpinocembrin, ficuformodiol A, ficuformodiol B, (R)-(−)-mellein, obovatin, spatheliachromene, 3-(7-methoxy-2,2-dimethyl-2H-6-chromenyl)-(E)-propenoic acid, chromenylacrylic acid, psoralen, β-sitosterol, stigmasterol, carpachromene, kaempferol, umbelliferone, and syringic aldehyde [10,11,12,13]. FF is traditionally used for treating osteoporosis in Southeast Asia and Taiwan. Furthermore, previous studies have reported that FF possesses anti-inflammatory properties [13], antioxidant properties [11,13], hepato-protective effects [14], and antitumor activities [10,11]. However, FF effects on DPN has received limited research attention. This study aimed to investigate the effect of FF extract (FFE) on DPN in ovariectomized diabetic mice.

## 2. Results

### 2.1. The Yield of FFE and Polyphenols and Flavonoids in FFE

The FFE yield from FF was 5.94% ± 0.45%. In this study, the FFE used was identified to contain polyphenols (73.43 ± 0.74 mg/g of gallic acid) and flavonoids (3.58 ± 1.13 mg/g of quercetin equivalent).

### 2.2. The Effect of FFE on Blood Glucose Levels in DM Mice

This study induced DPN in ovariectomized mice using STZ and a high-fat diet. Diabetic mice (DM group) had fasting blood glucose levels of 293.0 ± 22.3 mg/dL, indicating a successful DM induction. After the 6-week treatment, the fasting blood glucose levels in both the HFFE and LFFE groups were significantly reduced compared with those in the DM group (Table 1).

### 2.3. The Effect of FFE on DM Mice Behaviors

**Figure 1:**
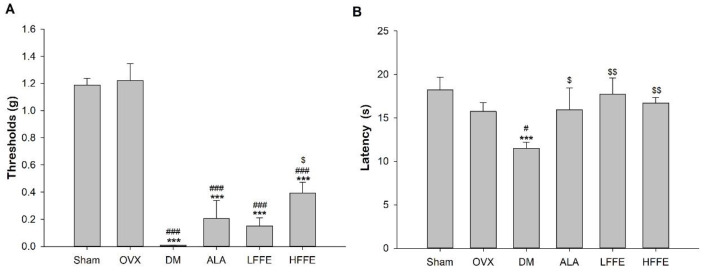
Mechanical paw withdrawal thresholds in response to stimulation with flexible von Frey filaments (**A**) and paw withdrawal latencies in response to thermal noxious stimuli (**B**) of high-fat diet/streptozotocin (HFD/STZ)-induced ovariectomized diabetic mice fed with *Ficus formosana* extract (FFE) for 6 weeks. Abbreviations are the same as those in Table 1. Each value is the mean ± SEM. *** *p* < 0.001 vs. sham group; ^#^ *p* < 0.05, ^###^ *p* < 0.001 vs. OVX group; ^$^ *p* < 0.05, ^$$^ *p* < 0.01 vs. DM group. N = 6 for each group.

The results of the von Frey filament test are presented in Figure 1A. The sham and OVX groups exhibited foot shrinkage, foot licking, and other reactions in response to mechanical stimulation thresholds. Conversely, the DM group responded to a significantly lower force than both the sham and OVX groups (*p* < 0.001). Moreover, the HFFE group had significantly increased thresholds, indicating alleviation of the hypersensitivity in the diabetic mice (*p* < 0.05).

In the thermal hyperalgesia test, the sham and OVX groups demonstrated a higher latency time than the DM group (*p* < 0.05). Furthermore, both the LFFE and HFFE treatments significantly increased thermal response times, indicating increased heat sensitivity in the diabetic mice (*p* < 0.05) (Figure 1B).

### 2.4. The Effect of FFE on Foot Epidermal Nerve Distribution in DM Mice

**Figure 2:**
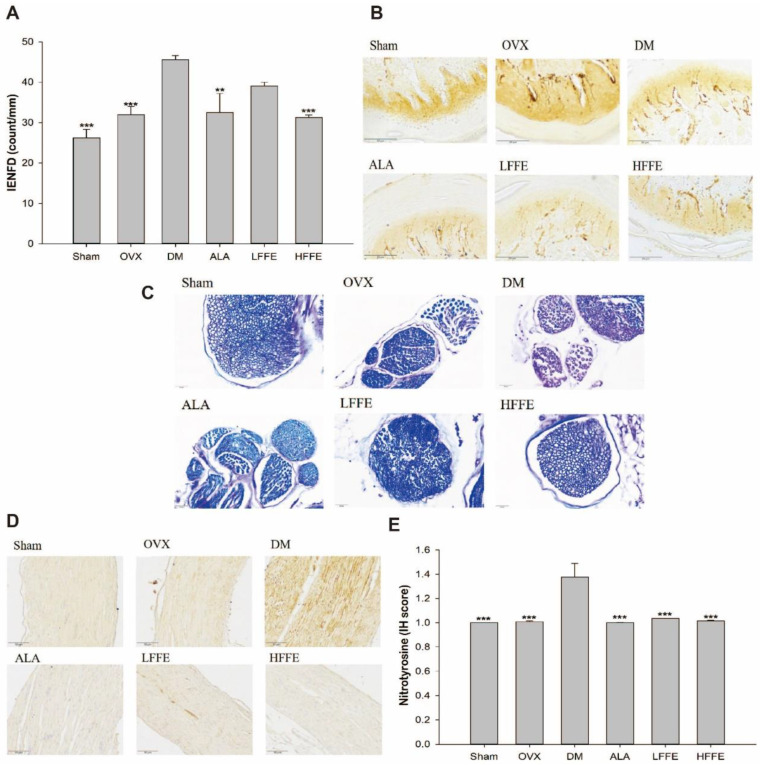
Intraepidermal nerve fiber density (**A**) and profiles (200×) (**B**) in the paw skin, myelin sheath biopsy of the sciatic nerve with Luxal fast blue stain (400×) (**C**), immunohistochemistry of nitrotyrosine in the sciatic nerves (200×) (**D**), and quantification (**E**) of HFD/STZ-induced ovariectomized diabetic mice fed with FFE for 6 weeks. Abbreviations are the same as those in Table 1. Each value is the mean ± SEM. ** *p* < 0.01, *** *p* < 0.001 vs. DM group. N = 6 for each group.

The DM group had significantly increased intraepidermal nerve fiber density (IENFD) levels compared with the sham and OVX groups, indicating increased nerve C fiber proliferation in the diabetic mice (*p* < 0.001). However, the HFFE treatment significantly reduced the C fiber hyperplasia in the diabetic mice (*p* < 0.01) (Figure 2A). Additionally, the nerves in the DM group exhibited more rupture, discontinuity, and hyperplasia than those in the sham or OVX groups. Moreover, both LFFE and HFFE treatments demonstrated a significant improvement in nerve rupture in the diabetic mice (*p* < 0.01) (Figure 2B).

### 2.5. The Effect of FFE on Myelin and Nerve Fibers in DM Mice

The DM group exhibited a lower blue positive reaction and an overall reddish-purple color compared to the sham and OVX groups, indicating a lower myelin content, looser nerve arrangement, and nerve damage in the diabetic mice (*p* < 0.05) (Figure 2C). Following LFFE and HFFE treatments, the myelin color turned blue, reflecting an increase in myelin content and denser nerve arrangement in the diabetic mice (Figure 2C).

### 2.6. The Effect of FFE on Nitrotyrosine Level in DM Mice

The DM group exhibited a darker stain color in tissue sections (reflecting a higher level of nitrotyrosine) than the sham and OVX groups, indicating severe oxidative damage in the diabetic mice (*p* < 0.001) (Figure 2D,E). However, both LFFE and HFFE treatments significantly decreased the nitrotyrosine level, indicating a reduction in tissue damage by ROS in the diabetic mice (*p* < 0.001) (Figure 2D,E).

### 2.7. The Effect of FFE on mRNA Expression of Genes Associated with Inflammation in DM Mice

**Figure 3:**
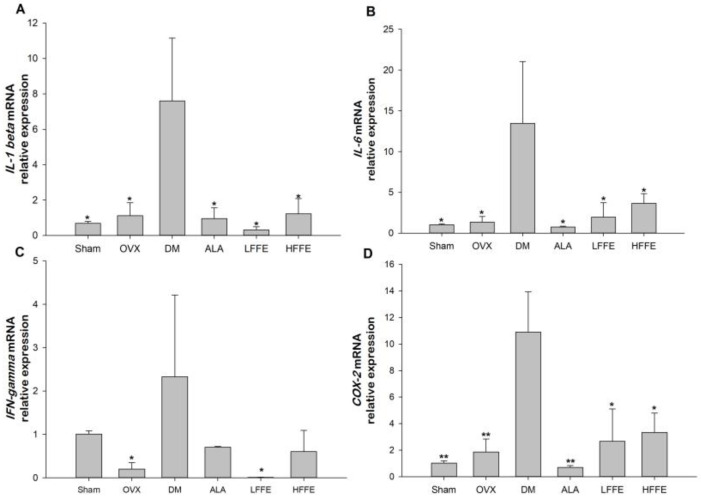
The messenger RNA expression of interleukin-1β (IL-1β, (**A**)), interleukin-6 (IL-6, (**B**)), interferon-γ (IFN-γ, (**C**)), and cyclooxygenase-2 (Cox-2, (**D**)) in the sciatic nerves of HFD/STZ-induced ovariectomized diabetic mice fed with FFE for 6 weeks. Abbreviations are the same as those in Table 1. Each value is the mean ± SEM. * *p* <0.05, ** *p* <0.01 vs. DM group. N = 6 for each group.

The interleukin (IL)-1β mRNA expression in the DM group was significantly increased compared with that in the sham and OVX groups (*p* < 0.05) (Figure 3A). LFFE and HFFE treatments significantly reduced IL-1β mRNA expression in the diabetic mice (Figure 3A). Furthermore, the DM group had enhanced IL-6 mRNA expression; however, it was significantly decreased by LFFE and HFFE treatments (Figure 3B). The interferon (IFN)-γ mRNA expression in the DM group was increased compared with that in the OVX group (*p* < 0.05) (Figure 3C). However, the LFFE treatment significantly reduced the IFN-γ mRNA expression in the diabetic mice (Figure 3C). Moreover, the DM group had significantly higher cyclooxygenase (COX)-2 mRNA expression than the OVX group (*p* < 0.05); however, both HFFE and LFFE treatments significantly decreased the COX-2 mRNA level in the diabetic mice (Figure 3D).

### 2.8. The Effect of FFE on of mRNA Expression of Genes Associated with Sodium Ion Channels in Inflammatory Pain in DM Mice

**Figure 4:**
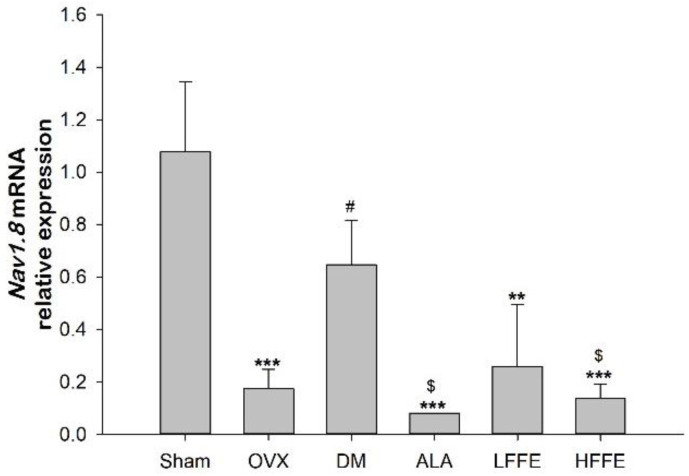
The messenger RNA expression of voltage-gated sodium channel 1.8 (Nav1.8) in the sciatic nerves of HFD/STZ-induced ovariectomized diabetic mice fed with FFE for 6 weeks. Abbreviations are the same as those in Table 1. Each value is the mean ± SEM. ** *p* < 0.01, *** *p* < 0.001 vs. sham group; ^#^ *p* < 0.05 vs. OVX group; ^$^ *p* < 0.05 vs. DM group. N = 6 for each group.

The DM group had significantly higher voltage-gated sodium channel (Nav) 1.8 mRNA expression than the OVX group (*p* < 0.05). However, the HFFE treatments significantly decreased voltage-gated sodium channel (Nav) 1.8 mRNA expression in the diabetic mice (*p* < 0.05) (Figure 4).

## 3. Discussion

In this study, a 6-week oral FFE administration decreased the blood glucose levels in ovariectomized diabetic mice. Further, FFE exhibited benefits in increasing neural response thresholds and alleviating pain responses, indicating amelioration of peripheral neuropathy in diabetic mice. The pathological examination also showed that FFE improved nerve proliferation and reduced axonal damage in diabetic mice. Excessive levels of inflammation-related factors including IL-1β and COX-2 in the sciatic nerve are associated with pain responses [15]. In this study, FFE administration decreased the mRNA expression of IL-1β, IL-6, IFN-γ, and COX-2, indicating the anti-inflammatory potential of FFE in ovariectomized diabetic mice.

Distal symmetrical neuropathy is one of the main DPN symptoms and is attributed to myelin that lacks C fibers, and is therefore particularly susceptible to free radical damage. This vulnerability results in peripheral nervous system impairment, and hyperglycemia-induced ROS exacerbate the paresthesias and nerve damage in diabetes [16]. In DPN mice, these fibers show abnormal sensitivity to temperature and touch and lead to nerve damage and numbness due to C fiber loss [17]. Sciatic nerve damage in ovariectomized diabetic mice may stem from an incomplete regenerative process and result in pain hypersensitivity [16,18]. Thermal hypersensitivity was observed, and FFE improved these symptoms in the ovariectomized diabetic mice in this study. Moreover, although peripheral sciatic nerves were damaged in diabetic mice, FFE reduced hyperplasia, enhanced C fiber morphology, and alleviated the abnormal sensitivity. This aligns with the behavioral trial results, indicating FFE’s potential in alleviating diabetic neuropathy symptoms.

Oxidative stress-induced poly ADP-ribose polymerase (PARP) activation has been linked to several diabetic complications, and PARP inhibitors have been reported to reduce nitrotyrosine production [19]. Nitrotyrosine serves as a pivotal biological marker for increases in cellular levels of ROS. This process, known as tyrosine nitration, involves the oxidation of the nitrogen atom on tyrosine, resulting in the formation of nitrotyrosine molecules. The detection of nitrotyrosine is commonly employed to assess the extent of oxidative stress and is recognized as a biomarker for cellular oxidative stress and oxidative damage [20]. In this study, FFE reduced nitrotyrosine production, suggesting a decrease in harmful reactive nitrogen species, including peroxynitrite and nitrogen dioxide, in the sciatic nerves of diabetic mice, and also suggests a decrease in ROS levels at the same time. In this study, FFE contained anti-oxidative phytochemicals, including polyphenols and flavonoids, which is consistent with the findings reported in previous studies [10,11,13]. We propose that the benefits of FFE in the present study are associated with these antioxidants. Hyperglycemia leads to pro-oxidative substance generation, results in oxidative stress and PARP activation, affects nuclear factor-κB activation, and subsequently leads to the secretion of inflammatory substances, including COX-2, IL-1β, and IL-6 by Schwann cells [4,21]. In this study, the mRNA expression levels of IL-6, IL-1β, and IFN-γ were increased in the diabetic mice, which is consistent with the results of previous studies.

Peripheral nerve damage may lead to the release of regulatory substances, such as glutamate, ATP, and bradykinin, which subsequently cause ectopic discharge. These substances facilitate nerve conduction and stimulate the secretion of inflammatory mediators by Schwann cells, reactivate neurons, and trigger a feedback cycle of inflammation and pain response. Additionally, the activation of certain pathways can increase the activity or expression of specific sodium channels and the transient receptor potential cation channel subfamily V member 1, thereby enhancing nerve conduction, reducing pain thresholds, and increasing cell excitability [4]. This leads to elevated peripheral nerve sensitization. Furthermore, increased levels of cytokines such as IL-1β may promote regeneration and suppress nerve apoptosis [22]. The activation of pathways including the Janus kinase/signal transducer and activator of transcription and extracellular signal-regulated kinase pathways may also contribute to the excessive pain sensation [23]. In this study, an increase in IL-1β mRNA expression and decrease in Nav1.8 levels were observed in diabetic mice. These findings suggest that abnormal pain responses are due to the enhanced ion channel activity and ectopic discharge in DM. Moreover, IL-1β has been reported to activate COX-2, subsequently leading to prostaglandin release and triggering nociceptor sodium channel phosphorylation in neuronal cells [24,25]. This increases peripheral sensitization and neuronal excitability, decreases pain thresholds, sensitizes peripheral nociceptor terminals, and localizes pain hypersensitivity. This phenomenon may contribute to the abnormal pain sensitivity in diabetic mice. In this study, FFE suppressed IL-1β and COX-2 mRNA expression, increased the mechanical and temperature stimulation thresholds, and reduced pain hypersensitivity, suggesting that FFE can improve DPN in diabetic mice.

In the present investigation, it is posited that the mitigation of DPN observed in the ovariectomized diabetic mice, attributable to FFE, is potentially correlated with its enriched content of polyphenols and flavonoids. Rigorous efforts are being made in our laboratory to accurately identify the active phytochemical constituents present in FFE, by leveraging advanced analytical methods such as column chromatography and high-performance liquid chromatography. Our observations align seamlessly with the previous research, exemplified by the study conducted by Pwaniyibo et al. [26], which demonstrated the inherent antidiabetic efficacies prevalent in an array of *Ficus* species, thereby alluding to a plausible variation in bioactive compounds (flavonoids, glycosides, alkaloids, saponins, tannins, terpenes, carotenoids, and steroids) contingent upon the specific species. Notwithstanding our primary emphasis on polyphenols and flavonoids, the research delineated by Din et al. [27] broadened the academic discourse by elucidating the antidiabetic attributes of a specific triterpene lactone, sourced from *Ficus*, thereby highlighting the expansive array of therapeutic compounds within this genus. Concurrently, Lin et al. [28] provided insights into an alternative extraction modality for *Ficus carica* leaves to alleviate diabetic mice and raise psoralen and umbelliferone which have substantial glucose-lowering activity. Collectively, the *Ficus* genus stands out as an invaluable repository of prospective antidiabetic compounds, with each species possessing unique medicinal properties.

In this study, there are some limitations to consider. Firstly, although we have identified polyphenols and flavonoids in the FFE as potential bioactive compounds, the specific active compounds remain an ongoing area of research. Further exploration of these compounds and their individual contributions to the observed effects is needed for a more comprehensive understanding. Secondly, while we used markers like nitrotyrosine to assess oxidative damage, a direct measurement of ROS levels was not conducted. Direct quantification of ROS could have provided more precise insights into the oxidative stress response. Future studies may benefit from including direct ROS measurement techniques to enhance our findings.

## 4. Materials and Methods

### 4.1. Preparation of FF

FF stem slices were purchased from Jiatai Qingcaohang (Chiayi City, Taiwan) in January 2019. The slices were boiled in water (FF, water = 1:10, *w*/*w*) for 3 h, filtered using a double-layer gauze, and subsequently freeze-dried to obtain FFE samples [13]. A voucher specimen (TaiBNET204841) has been deposited in the herbarium of Biodiversity Research Center, Academia Sinica, Taiwan.

### 4.2. Analysis of Total Polyphenol and Flavonoid in FFE

A 0.1 mL of 10,000 ppm (10 mg/mL) FFE was mixed with 2 mL of 2% Na_2_CO_3_ for 2 min, followed by reaction with 0.1 mL of 50% Folin–Ciocalteu reagent in the dark at 25 °C for 30 min, and the absorbance was measured at 750 nm. Using the gallic acid calibration curve, the polyphenol content was determined and expressed as mg/g of gallic acid [29]. Subsequently, 0.5 mL of 10,000 ppm (10 mg/mL) FFE was mixed with 1.5 mL ethanol, 0.1 mL 10% aluminum chloride, 0.1 mL 1 M potassium acetate, and 2.8 mL dd water; the mixture was incubated in the dark for 30 min; and the absorbance was measured at 415 nm. The flavonoid content was expressed as mg/g of quercetin equivalent [30].

### 4.3. Animal Experimental Procedures

Thirty-six eight-week-old female C57BL/6J mice (BioLASCO Co., Ltd., Taiwan, Taipei, Taiwan) were reared in a temperature (20 °C ± 2 °C)- and humidity (50% ± 20%)-controlled room with a 12 h light/dark cycle. A phytoestrogen-free normal diet or phytoestrogen-free 60% high-fat powdered diet (D10012G; Research Diets Inc., New Brunswick, NJ, USA) and sterile water were offered ad libitum. The experimental design is shown in Supplementary Figure S1.

Six mice were subjected to sham surgery, whereas the others underwent ovariectomy (OVX). During the recovery period, the mice were fed standard chow for 2 weeks. In the third week, to induce DM, 24 mice that underwent OVX were intraperitoneally injected with streptozotocin (STZ, 50 mg/kg BW) for five consecutive days [31]. These DM mice were subsequently orally administered low-dose FFE (20 mg/kg BW, LFFE), high-dose FFE (200 mg/kg BW, HFFE), and alpha-lipoic acid (250 mg/kg BW, ALA, positive control group) once daily for 6 weeks. All mice were fasted overnight and subsequently sacrificed at the end of the experiment. The serum glucose level was determined with an enzymatic kit (Crumlin Co., Antrim, UK).

### 4.4. Von Frey Filament Test

To minimize experimental errors caused by agitation and anxiety, the mice were acclimated on a fine-mesh test bench with the lid covered by tape for 15 min. To stimulate the right hind paw of the mice, the von Frey filament test with different gram forces was performed, and their responses including shrinkage, licking, or foot monitoring were subsequently observed. If responses were not observed, a higher gram force stimulus would be applied. The threshold for response was provided by calculating the experimental relative code [32].

### 4.5. Thermal Hyperalgesia Test

Before the test, to reduce their anxiety, the mice were placed in an acrylic material (0.3 cm thickness) device (20 × 20 × 20 cm^3^) at room temperature for 15 min once a week for 3 weeks. During the trial, the device temperature was set to 52 °C in a constant-temperature water bath. Once the hind paw of mice touched the bottom of the device covered with aluminum foil, the time was recorded until they exhibited licking, shaking, jumping, or looking at their feet.

### 4.6. Collection of Sciatic Nerve and Paw Tissues

Mice were fasted for 8–12 h before sacrifice. They were euthanized with 250 mg/kg BW Avertin (Merck, Darmstadt, Germany) by intraperitoneal injection. Both the sciatic nerves of the hind legs and right hind paw were removed and stored at −80 °C or in 10% formalin at room temperature.

### 4.7. Quantification of Epidermal Nerve Fiber Density

The upper edge of the leg and paw epidermis was measured using viewpoint light software (DxO ViewPoint Version 1.0.0.9628). The epidermal nerve fiber density was calculated based on the number of nerves per millimeter [33].

### 4.8. Immunohistochemistry

The paw was longitudinally cut into 9 μm paraffin sections, and oven-dried at 65 °C for 30 min. After 15 min of dewaxing with xylene (Amos Scientific Pty, Ltd., Taoyuan, Taiwan) and rehydration in decreasing ethanol concentrations, the samples were exposed to a 15-fold diluted citrate buffer for 20 min. The tissues were drip washed by blocking buffer for 10 min and reacted with 1:200 anti-PGP9.5 as the first antibody at 4 °C overnight. Subsequently, anti-rabbit IgG was as the secondary antibody and reacted for 1 h at room temperature, followed by staining with 3,3′-diaminobenzidine and oven-drying. The slides were mounted and observed using an optical microscope. A similar protocol was used for assessing the nitrotyrosine level in the sciatic nerve, which was embedded in paraffin wax and sliced into 3 mm thick sections. Next, 1:200 anti-nitrotyrosine (Cell Signaling Technology, Danvers, MA, USA) was used as the first antibody, followed by a 30 min secondary antibody reaction. Quantitation of the immunostaining was performed using the Image Java software (Version 1.53a). The immunohistochemistry scores for each tissue section were quantified using the immunohistochemistry Profiler macro plugin, with scores categorized into four levels: 1 for negative, 2 for low positive, 3 for positive, and 4 for high positive.

### 4.9. Luxal Fast Blue Staining of Sciatic Nerve Myelin

The paws of mice were cut vertically at a thickness of 3 μm and then embedded in paraffin wax. The staining procedure was adapted from a previous study [34].

### 4.10. Real-Time Quantitative PCR Analysis

Total RNA extraction from the sciatic nerve was performed using a published protocol [35]. Briefly, sciatic nerve tissues were homogenized with REzol C&T RNA extraction reagent in an ice bath, mixed with the RNA separation solution, and centrifuged to obtain the supernatant. The supernatant was added to isopropanol and then centrifuged. The pellet was collected and rinsed with ethanol; then, the solvent was removed using a rotary evaporator to obtain the RNA. The RNA was reverse-transcribed into cDNA using the MMLV reverse transcription kit. To obtain the template and primer premix, oligodT was added to the solution was added and heated in Bio-Rad MyCycler 580BR 96-Well PCR Thermal Cycler. The premix was treated with a reaction buffer, RNase inhibitor, dNTP premix, and MMLV reverse transcriptase, and reheated to obtain template cDNA. The template cDNA was subsequently mixed with qPCRBIO SyGreen Blue Mix, forward primer, reverse primer, and DEPC water. The gene expression was analyzed using a real-time PCR detection system and SuperReal PreMix Plus kit. The relative mRNA expression levels of the target genes, including IL-1β, IL-6, IFN-γ, COX-2, Nav 1.8, and β-actin, were calculated using the 2^−△△Ct^ method, and the specific primer sequences used for amplification are shown in Table 2.

### 4.11. Statistical Analyses

The data analysis was conducted using SPSS version 23.0 (SPSS Inc., Chicago, IL, USA) and a one-way analysis of variance, followed by post hoc Duncan’s multiple range test. The data are presented as mean ± standard error of the mean. Statistical significance was defined as *p* < 0.05.

## 5. Conclusions

**Figure 5:**
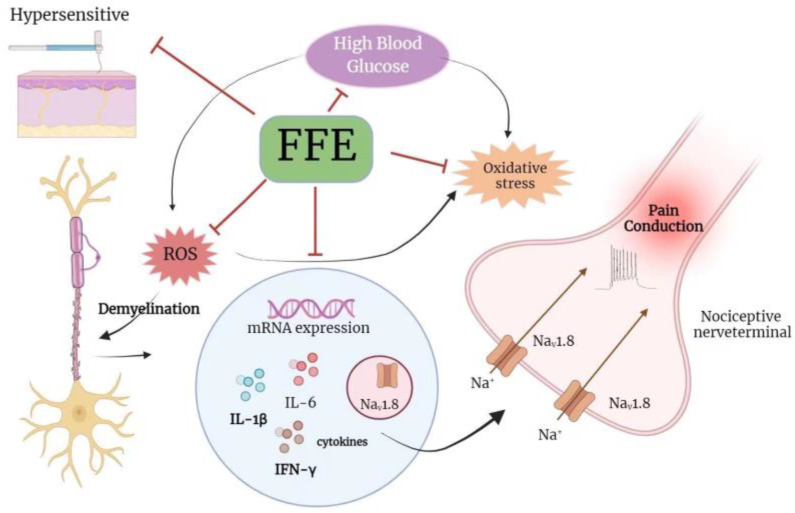
The possible mechanism of FFE in ameliorating diabetic peripheral neuropathy in ovariectomized diabetic mice. (Created in BioRender.com).

This study demonstrated the ameliorative effect of FFE on DPN in ovariectomized diabetic mice. The results suggest that FFE protects the sciatic nerve via reducing oxidative stress and suppressing the expression of inflammatory genes, including IL-1β, IL-6, and IFN-γ, in diabetic mice. Additionally, FFE may inhibit sodium ion channel overactivation, thereby alleviating peripheral neuron cell pain transmission in diabetic mice (Figure 5). This protective effect may be attributed to the potent antioxidants in FFE, including polyphenols and flavonoids. Further analysis to purify and identify the bioactive components in FFE for a more comprehensive understanding of its mechanism is currently underway in our laboratory.

## Figures and Tables

**Table 1:** The blood glucose in HFD/STZ-induced ovariectomized diabetic mice fed with FFE for 6 weeks.

	Sham	OVX	DM	ALA	LFFE	HFFE
Fasting blood glucose (mg/dL)	119.2 ± 3.0	115.3 ± 1.7	268.3 ± 53.6 *^#^	244.8 ± 12.7 *^#^	225.5 ± 39.3	212.8 ± 33.0

Sham: sham mice fed with normal diet. OVX: ovariectomized mice fed with normal diet. DM: OVX mice treated with HFD (60% fat diet) and STZ injection to induce diabetes. ALA: DM mice gavaged with alpha-lipoic acid 250 mg/kg BW/day and fed with HFD. LFFE: DM mice gavaged with FFE 20 mg/kg BW/day and fed with HFD. HFFE: DM mice gavaged with FFE 200 mg/kg BW/day and fed with HFD. Each value is the mean ± SEM. * *p* < 0.05 vs. sham group; ^#^ *p* < 0.05 vs. OVX group. N = 6 for each group.

**Table 2:** Primer sequences for RT-PCR.

Primer	Forward	Reverse
β-actin	5′-GTGACGTTGACATCCGTAAAGA-3′	5′-GCCGGACTCATCGTACTCC-3′
IFN-γ	5′-CGGCACAGTCATTGAAAGCC-3′	5′-TGTCACCATCCTTTTGCCAGT-3′
IL-6	5′-GCCTTCTTGGGACTGATG-3′	5′-AGGTCTGTTGGGAGTGGTA-3′
IL-1β	5′-GAAATGCCACCTTTTGACAGTG-3′	5′-TGGATGCTCTCATCAGGACAG-3′
COX-2	5′-TGTCACCATCCTTTTGCCAGT-3′	5′-GCTCGGCTTCCAGTATTGAG-3′
Nav1.8	5′-AATCAGAGCGAGGAGAAGACG-3′	5′-CTAGTGAGCTAAGGATCGCAGA-3′

## Data Availability

Data are available in a publicly accessible repository.

## Supplementary Materials

The following supporting information can be downloaded at: https://www.mdpi.com/article/10.3390/plants12213774/s1, **Figure S1**: Experimental design of this study. Sham: Sham mice fed with normal diet. OVX: Ovariectomized mice fed with normal diet. DM: OVX mice treated with high-fat diet (HFD; 60% fat diet) and streptozotocin (STZ) injection to induce diabetes and fed with HFD. ALA: DM mice gavaged with alpha-lipoic acid 250 mg/kg BW/day and fed with HFD. LFFE: DM mice gavaged with *Ficus formosana* extract (FFE) 20 mg/kg BW/day and fed with HFD. HFFE: DM mice gavaged with FFE 200 mg/kg BW/day and fed with HFD. PCR: polymerase chain reaction
