# Supplementary material for: Alleviative Effect of Ficus formosana Extract on Peripheral Neuropathy in Ovariectomized Diabetic Mice
Source: Plants (Basel). 2023 Nov 5;12(21):3774. doi: 10.3390/plants12213774 (PMC10649879; doi:10.3390/plants12213774)
Supplement: Supplementary file 1 [file plants-12-03774-s001.zip › plants-2636604-supplementary.pdf]

plants

MDPI

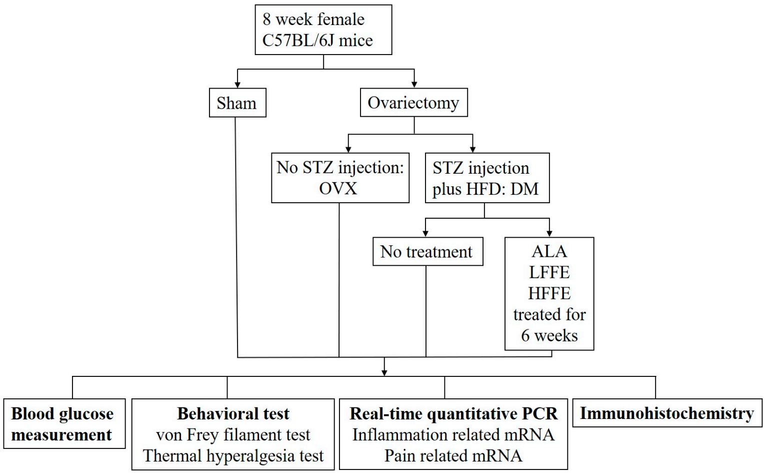


**Supplementary Figure S1.** Experimental design of this study. Sham: Sham mice fed with normal diet. OVX: Ovariectomized mice fed with normal diet. DM: OVX mice treated with high-fat diet (HFD; 60% fat diet) and streptozotocin (STZ) injection to induce diabetes and fed with HFD. ALA: DM mice gavaged with alpha-lipoic acid 250 mg/kg BW/day and fed with HFD. LFFE: DM mice gavaged with *Ficus formosana* extract (FFE) 20 mg/kg BW/day and fed with HFD. HFFE: DM mice gavaged with FFE 200 mg/kg BW/day and fed with HFD. PCR: polymerase chain reaction.
